# Supplementary figures and images for: Flavivirus NS1-triggered endothelial dysfunction promotes virus dissemination
Source: PLoS Pathog. 2025 Dec 31;21(12):e1013811. doi: 10.1371/journal.ppat.1013811 (PMC12779140; doi:10.1371/journal.ppat.1013811)

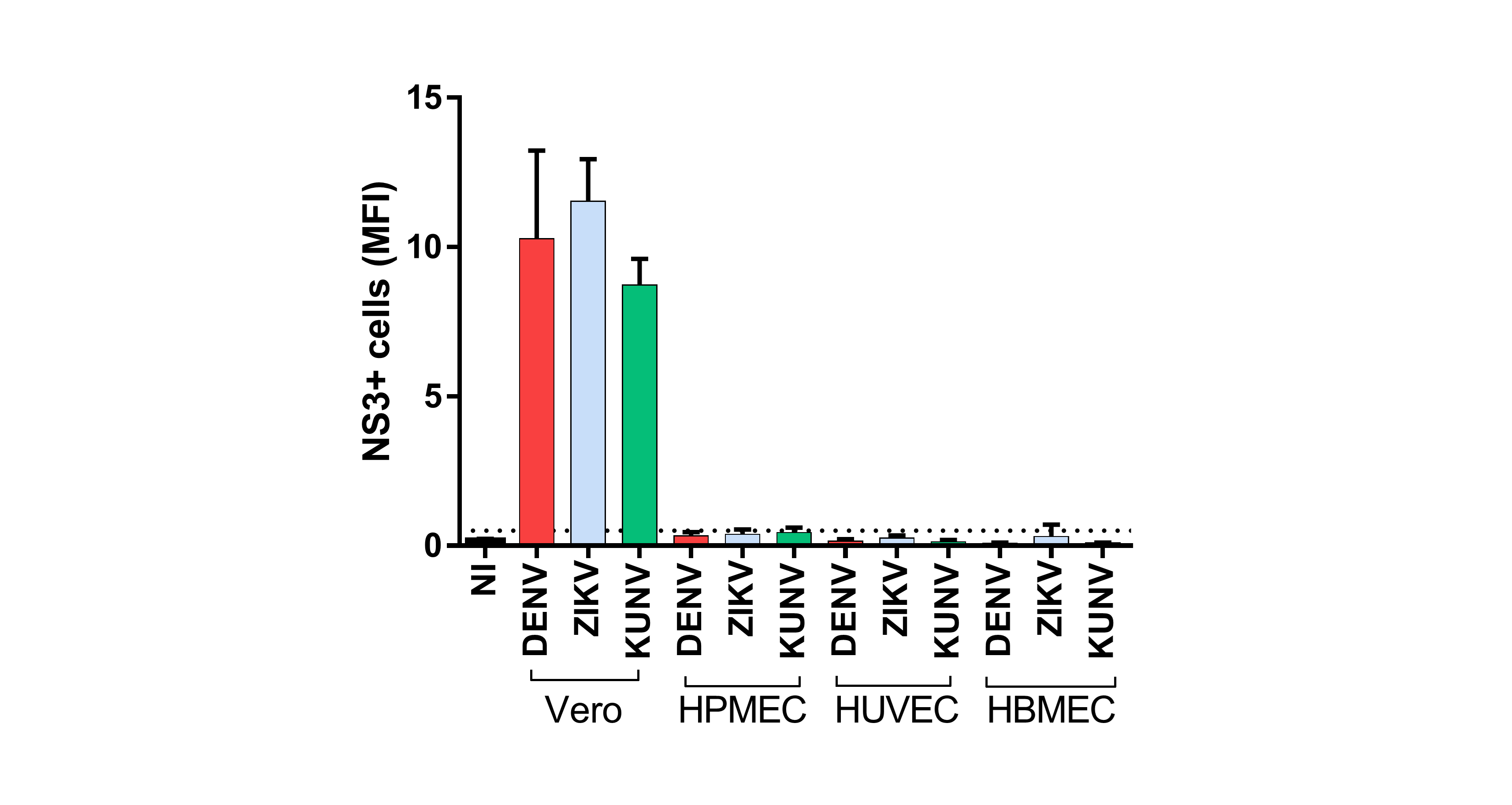

Supplement: S1 Fig — Monolayers of HPMEC, HUVEC, HBMEC, or Vero-CCL81 cells grown on transwells were treated for 3 hours with the indicated NS1 proteins (5 µg/mL). Cells were then treated with 1x10^5^ FFU of DENV, 1x10^5^ FFU of ZIKV, or 1x10^6^ FFU KUNV (added to the apical chamber) as in Figs 2 and 3. Twenty-four hours later, cells were fixed in the transwells, and viral infection was monitored by IFA staining for NS3. Quantification of mean fluorescence intensity (MFI) was normalized to cell number. (TIF) [file ppat.1013811.s001.tif]

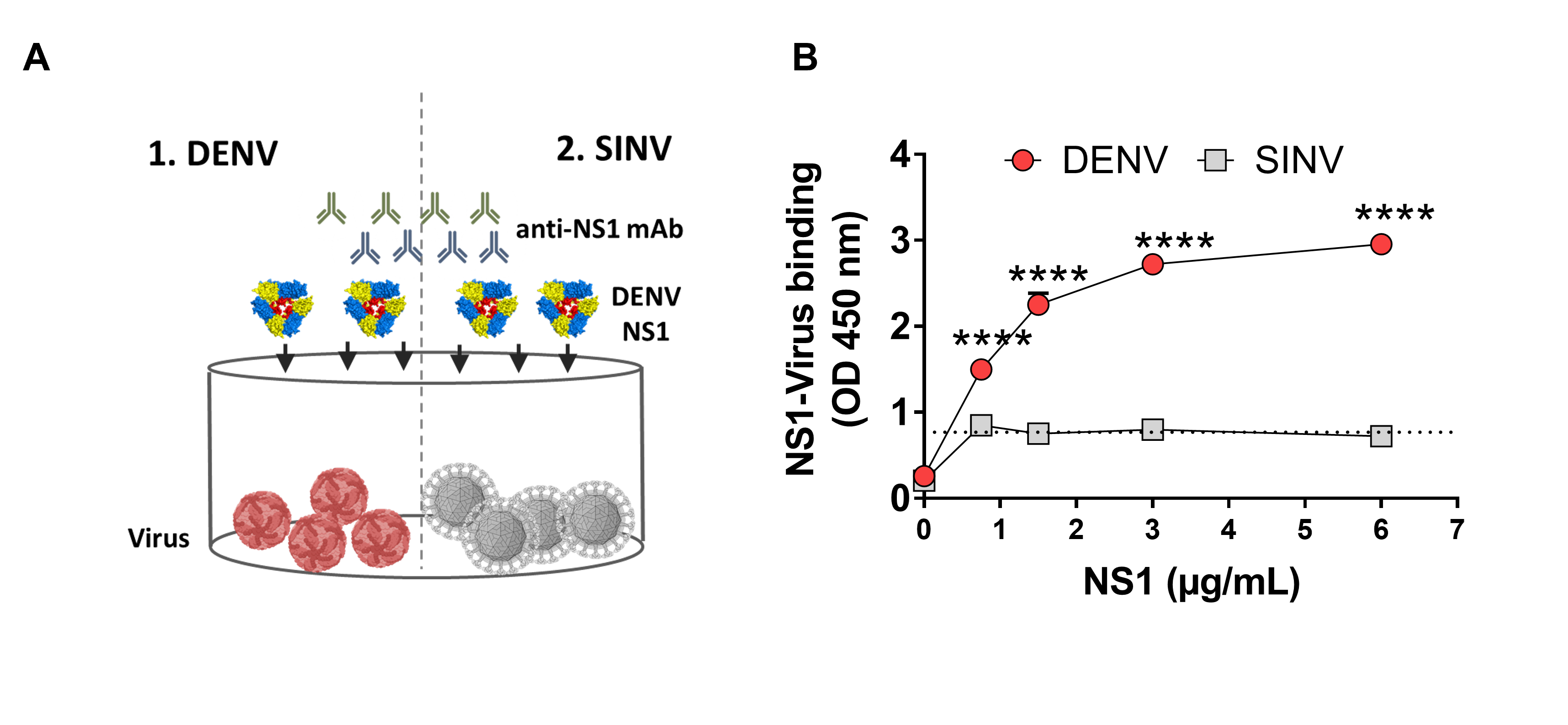

Supplement: S2 Fig — (A and B) ELISA plates were coated with 1x10^8^ viral particles of (1) DENV or (2) Sindbis virus (SINV). Then, the indicated concentration of DENV NS1 was added, captured by immobilized virions, and detected using an anti-NS1 antibody. Captured NS1 was measured by absorbance at 450 nm. Results are depicted from 3 independent experiments. Two-way ANOVA with multiple comparisons showed statistical significance with **** p < 0.0001. Created in part using BioRender. Harris, E. (2025) https://BioRender.com/k25vqzb. (TIF) [file ppat.1013811.s002.tif]

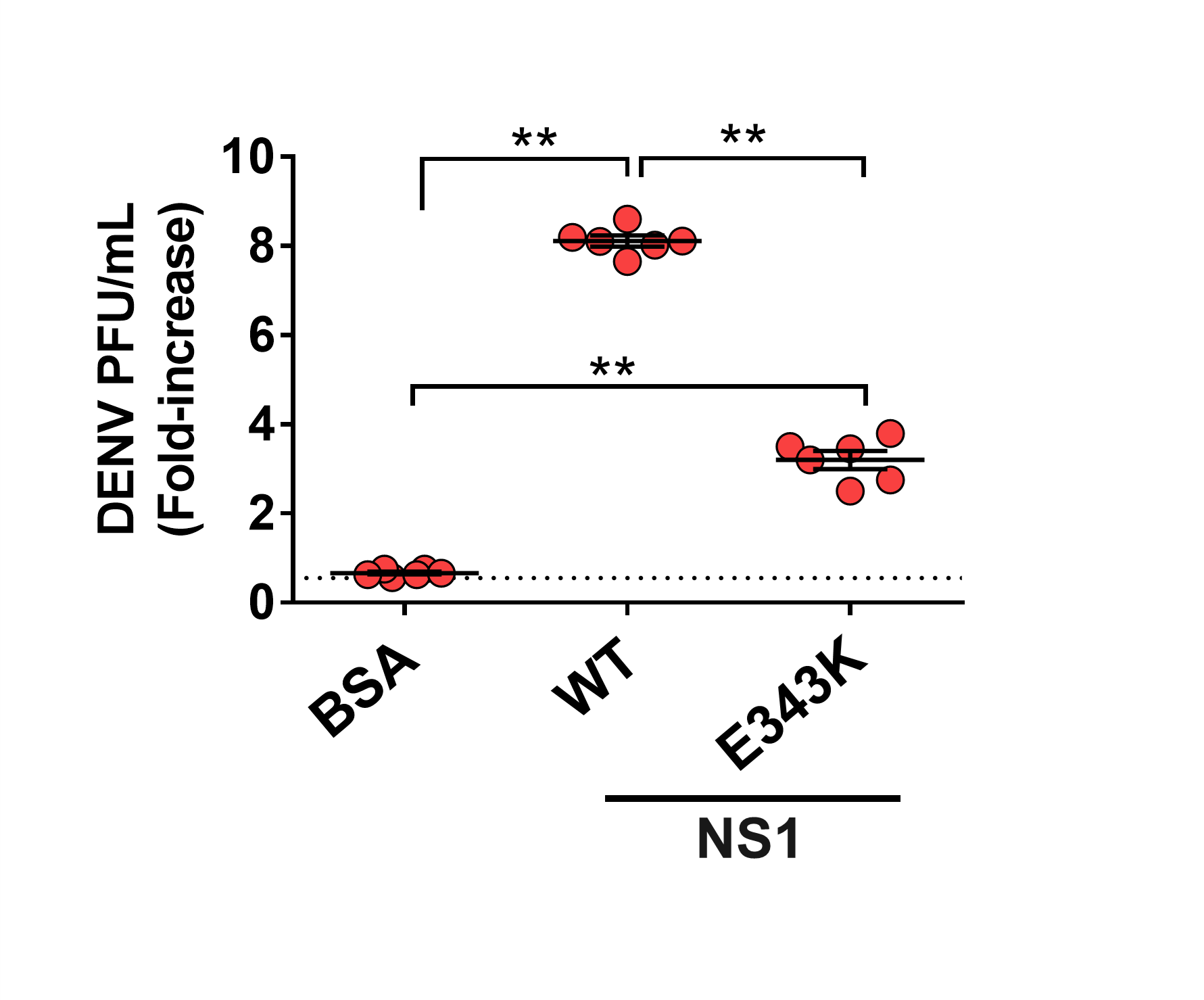

Supplement: S3 Fig — DENV titers (PFU/mL) were measured after infection of BHK cells in the presence of BSA, NS1 WT, or the NS1 E343K mutant. BSA was used as a protein control and is indicated by the dotted line. Data represent the mean fold-increase of DENV titers in each condition compared to virus alone + /- the standard error of the mean, with at least three biological replicates. Groups indicated by brackets were compared using the non-parametric Mann-Whitney test. with **p < 0.01. (TIF) [file ppat.1013811.s003.tif]
